# Anthropogenic and natural drivers of gene flow in a temperate wild fruit tree: a basis for conservation and breeding programs in apples

**DOI:** 10.1111/eva.12250

**Published:** 2015-02-25

**Authors:** Amandine Cornille, Alice Feurtey, Uriel Gélin, Jeanne Ropars, Kristine Misvanderbrugge, Pierre Gladieux, Tatiana Giraud

**Affiliations:** 1 Ecologie, Systématique et Evolution, Université Paris-SudOrsay, France; 2 CNRSOrsay, France; 3 Department of Plant Ecology and Evolution, Uppsala UniversityUppsala, Sweden; 4 Département de biologie, Université de SherbrookeSherbrooke, QC, Canada; 5 Institute for Forestry and Game ManagementGeraardsbergen, Belgium

**Keywords:** admixture, crabapple, dispersal, glacial refugia, global changes, pollinators, SPIPOLL

## Abstract

Gene flow is an essential component of population adaptation and species evolution. Understanding of the natural and anthropogenic factors affecting gene flow is also critical for the development of appropriate management, breeding, and conservation programs. Here, we explored the natural and anthropogenic factors impacting crop-to-wild and within wild gene flow in apples in Europe using an unprecedented dense sampling of 1889 wild apple (*Malus sylvestris*) from European forests and 339 apple cultivars (*Malus domestica*). We made use of genetic, environmental, and ecological data (microsatellite markers, apple production across landscapes and records of apple flower visitors, respectively). We provide the first evidence that both human activities, through apple production, and human disturbance, through modifications of apple flower visitor diversity, have had a significant impact on crop-to-wild interspecific introgression rates. Our analysis also revealed the impact of previous natural climate change on historical gene flow in the nonintrogressed wild apple *M. sylvestris*, by identifying five distinct genetic groups in Europe and a north–south gradient of genetic diversity. These findings identify human activities and climate as key drivers of gene flow in a wild temperate fruit tree and provide a practical basis for conservation, agroforestry, and breeding programs for apples in Europe.

## Introduction

Over the last century, researchers in evolutionary and applied biology have paid much attention to gene flow, which is considered to play a key role in species persistence, evolution, and diversification. Many studies have investigated the consequences of intra- and interspecies gene flow for evolution (e.g., Garant et al. 2007; Feder et al. 2012). However, the anthropogenic and natural determinants of gene flow remain largely unexplored (Manel et al. 2003; Manel and Holderegger 2013) because multiple data sources (genetic, environment, ecology) are required to unravel these issues. One central question concerns the extent to which climate changes and recent global changes due to human disturbances (e.g., land-use management and fragmentation) affect gene flow (Manel and Holderegger 2013). This question must be addressed, to make it possible to forecast the impact of these changes on the evolution of populations and to develop appropriate management, breeding, and conservation programs.

Within-species gene flow, which is mediated by pollen and seed dispersal, is a key factor underlying population persistence (Ellstrand 2014). Gene flow shapes the spatial genetic structure and can therefore be inferred from the amount of genetic differentiation between populations by the use of neutral marker loci (Vekemans and Hardy 2004). Over the last 20 years, an array of methods, including autocorrelation, parentage analyses, and assignment methods, has been developed for the identification of natural factors affecting gene flow (Pritchard et al. 2000; Manel et al. 2003; Smouse and Sork 2004; Storfer et al. 2010). Studies based on these methods have identified climate as a key driver of within-species historical gene flow in many temperate species in Europe during the Pleistocene (Hewitt 2004). In particular, climate-driven range fluctuations during the Pleistocene have continuously reshaped genetic structure within species. However, the impact of climate on gene flow remains largely unexplored in scattered species, because of the difficulties of large-scale sampling. Yet, the determination of genetic diversity, differentiation, and admixture across the geographical range of such scattered species can be a practical basis for sustainable management and conservation programs.

Human activities also affect gene flow. In particular, the anthropization of landscapes and the cultivation of crops over extended areas have increased the opportunities for contact between cultivated plants and their wild relatives. Over the last 20 years, crop-to-wild gene flow has been considered a human disturbance and has received close attention, particularly in the context of the cultivation of transgenic crops and the ecological consequences of such gene flow for the fitness and genetic integrity of wild relatives (Ellstrand et al. 1999; Ellstrand 2003). Crop-to-wild gene flow has been investigated using neutral markers and shown to be widespread (Ellstrand et al. 1999; Coart et al. 2003; Delplancke et al. 2011; Aerts et al. 2013). However, only a few studies (Arnaud et al. 2003) have investigated the human disturbances and anthropogenic landscape drivers affecting crop-to-wild gene flow, despite the potentially large number of such drivers at work. Local human food crop production can affect crop-to-wild gene flow directly, by modifying the distribution and abundance of the crop. Humans can also have indirect effects on introgression rates, through their land-use practices. For instance, clear evidence has been obtained of a decline in the populations of both wild and domesticated pollinators associated with the intensification of cultivation and landscape management (Kremen et al. 2007). Pollinators are key vectors of gene flow and allele distribution in landscapes. Land-use changes can therefore have also an indirect impact on both crop-to-wild and within wild gene flow, through anthropogenic effects on the distribution and movements of the pollinator in the landscape.

Fruit trees, particularly scattered trees, are key species in ecosystems (Manning et al. 2006), and fruit tree crops are essential to human society, due to their contribution to worldwide food production (e.g., apple, grape, orange, coffee, cocoa, olive). However, despite the ecological and economic importance of these trees, much uncertainty remains concerning the impact of recent global changes on gene flow from crop-to-wild species and within wild species, particularly in temperate fruit trees. The cultivated apple (*Malus domestica*) is the one of the most important fruit tree crop in terms of worldwide production (http://faostat.fao.org/). It was introduced into Europe 1500 years ago and has been subject to the introgression of genes from the local wild European apple, *Malus sylvestris,* to the extent that *M. domestica* is now more closely related to *M. sylvestris* than to its primary progenitor in the Tian Shan, *M. sieversii* (Cornille et al. 2012). Reciprocally, crop-to-wild introgressions have been reported in the European wild apple *M. sylvestris* (Coart et al. 2006; Larsen et al. 2006; Gross et al. 2012; Cornille et al. 2013b). Investigations of the demographic history of the European wild apple have revealed the existence of three main genetic groups shaped by past climate change during the Pleistocene (Cornille et al. 2013a). However, both a sampling gap in the distribution of *M. sylvestris* in France and a lack of information about the pollination ecology of this species have limited our understanding of the structure of the population and the identification of drivers of intra- and interspecies gene flow. Yet, cultivated and wild apples in Europe, with their high levels of gene flow from crop-to-wild species and within wild species, easily detectable due to relatively recent secondary contact, constitute an ideal model for investigations of the anthropogenic and natural drivers of gene flow in fruit trees at large geographic scales.

We report here an unprecedented dense sampling of *M. sylvestris* across Europe (1889 individuals), with a focus in France (1092 individuals), unique for a temperate fruit tree. We also sampled a large number of *M. domestica* varieties (339 varieties), and combined genetic (microsatellites), ecological, and environmental data, to investigate the natural and anthropogenic drivers of gene flow in apples. Using genetic data across Europe, we estimated the degree of crop-to-wild introgressions into the European wild apple, across its geographic distribution and investigated the impact of climate on population structure and diversity, through estimates of historical gene flow within the nonintrogressed wild apple gene pool. Furthermore, by estimating recent crop-to-wild introgressions in France, and using environmental and ecological data, we investigated the effects of apple production on these crop-to-wild introgressions. Finally, we determined whether human activities had indirectly impacted rates of gene flow through their effects on the flower visitor community.

Altogether, we highlighted the impact of previous natural climate change on historical gene flow across Europe in the wild apple and identify human activities as key drivers of gene flow in a wild temperate fruit tree. Our landscape genetics study provides useful information for *in situ* and *ex situ* conservation management for this emblematic fruit tree species in Europe, for agroforestry programs aiming at integrating wild apples in agrosystems and for future breeding programs, the European wild apple being a major contributor to the cultivated apple genome.

## Materials and methods

### Sampling, DNA extraction, and microsatellite genotyping

In addition to the 599 samples of *M. sylvestris* from Europe previously studied (Cornille et al. 2013a,b) (38 sites), we collected a further denser sample of 1290 *M. sylvestris* trees (28 sites), mostly in France (20 sites, *N *=* *1092), but also in Denmark (6 sites, *N *=* *162), Italy (1 site, *N *=* *5) and Belgium (1 site, *N *=* *31). In total, 1889 *M. sylvestris* trees from 66 sites were analyzed for this study. In addition to the 299 *M. domestica* previously analyzed (Cornille et al. 2012), we sampled additional *M. domestica* trees in the field in Denmark (*N *=* *24) and in French apple orchards (*N *=* *16). In total, we analyzed 339 *M. domestica* trees in this study. The dataset for this study thus corresponds to 2228 individuals (details of the samples are provided in Table S1 and Fig. S1).

DNA was extracted with the NucleoSpin® plant II DNA extraction kit (Macherey & Nagel, Düren, Germany). Multiplex microsatellite PCR amplifications were performed with a multiplex PCR kit (Qiagen Inc.) as previously described (Cornille et al. 2012; Patocchi et al. 2009), with 26 microsatellite markers. We retained only multilocus genotypes for which <20% of the data were missing. The suitability for population genetic analysis of the markers used was demonstrated in previous studies (Cornille et al. 2012, 2013a,b).

### Crop-to-wild gene flow, population structure, and historical gene flow (large-scale analyses, Europe)

#### Bayesian inference of crop-to-wild gene flow, population structure, and admixture

We used the individual-based Bayesian clustering methods implemented in STRUCTURE 2.3.3 (Pritchard et al. 2000) to estimate recent introgression from *M. domestica* into *M. sylvestris*. STRUCTURE uses Markov chain Monte Carlo (MCMC) simulations to infer the proportion of ancestry of genotypes from *K* distinct clusters. The underlying algorithms attempt to minimize deviations from Hardy–Weinberg within clusters and linkage disequilibrium among loci. A lack of consideration of intraspecies genetic structure in STRUCTURE analyses can bias the interpretation of introgression rates (Kalinowski 2011; Cornille et al. 2013b). We ran STRUCTURE, assuming *K *=* *1 to *K *=* *9 clusters, on the whole dataset including *M. sylvestris* (*N *=* *1889) and *M. domestica* (*N *=* *339). Based on 10 repeated runs of MCMC sampling comprising 500 000 iterations after a burn-in of 50 000 steps, we determined the amount of additional information explained by increasing *K,* using the Δ*K* statistic (Evanno et al. 2005). As a genetic structure was observed within the *M. sylvestris* dataset (see below), we assumed that the total membership coefficient of *M. sylvestris* individuals in the *M. sylvestris* gene pool was the sum of their membership coefficients in each *M. sylvestris* cluster. All *M. sylvestris* individuals with a cumulative membership coefficient for the *M. sylvestris* gene pool >0.9 were considered to be pure *M. sylvestris* trees. Conversely, all *M. sylvestris* individuals with a cumulative membership coefficient >0.1 in the *M. domestica* gene pool were considered to be admixed with *M. domestica*. *Malus sylvestris* individuals with a cumulative membership coefficient <0.1 in the *M. sylvestris* gene pool were considered to be misidentified individuals actually belonging to *M. domestica*. The proportion admixture of the *M. domestica* gene pool with the *M. sylvestris* gene pool is hereafter denoted *P*_DOM→SYL_ and referred as ‘introgression rate’. In the absence of genomewide genotyping data, we probably failed to identify advanced generation backcrosses. However, our approach aimed to identify early generations of admixture and introgression, because our focus here was the effect of recent human disturbance on gene flow.

To investigate the population structure and admixture of *M. sylvestris*, we excluded hybrids previously identified as displaying introgression from *M. domestica* (i.e.*,* individuals assigned to a wild gene pool with a membership coefficient <0.9; *N *=* *1376 individuals, 62 sites) and used the individual-based spatially explicit Bayesian clustering method implemented in TESS 2.3.1 (Chen et al. 2007). TESS is similar to STRUCTURE, except that it incorporates a spatial component into the clustering procedure, such that genotypes from areas located close together geographically are considered more likely to belong to the same cluster. We used the conditional autoregressive (CAR) Gaussian model of admixture with linear trend surface, setting the spatial interaction parameter (*ρ*) to 0.6. These parameters (*ρ* and trend) affect the weight given to spatial distance when clustering genotypes. Ten independent analyses were carried out for each number of clusters *K* (2 ≤ *K*_max_ ≤ 9), using 500 000 MCMC iterations after a burn-in of 50 000 steps. We determined the amount of additional information explained by increasing *K,* using the rate of change of the deviation index criterion (DIC) with increasing *K*.

For both TESS and STRUCTURE analyses, outputs were processed with CLUMPP v1.1.2 (Jakobsson and Rosenberg 2007), to identify distinct clustering solutions in the replicated runs for each *K* value.

#### Genetic variation within *M. sylvestris*

For the analysis of within-species genetic variation, we excluded hybrids identified as displaying introgression from *M. domestica*. Descriptive statistical analyses were performed for each site (i.e.*,* geographical location) and each population (i.e.*,* clusters inferred by TESS, including hybrids with a membership coefficient of up to 0.55 for the focal cluster).

Allelic richness (*A*_R_) and private allelic richness (*A*_P_) were calculated with ADZE (Szpiech et al. 2008), using standardized sample sizes of *N *=* *2 (one individual x two chromosomes) and *N = *100 (50 individuals × two chromosomes), respectively, corresponding to the minimal number of observations across sites and populations. Heterozygosities (expected and observed), Weir and Cockerham *F*-statistics and deviations from Hardy–Weinberg equilibrium were calculated with GENEPOP 4.2 (Raymond and Rousset 1995; Rousset 2008). Only sampling sites for which at least four individuals were successfully genotyped were included in site-specific calculations (51 of the 62 sites). Spatial patterns of genetic variability were visualized by mapping variation (*A*_R_), for 43 sites (i.e.*,* geographical locations with at least four individuals and for which at least two individuals were successfully genotyped for each marker) and genetic diversity (*H*_O_) for 51 sites across space, with the kriging and surface functions (*field* R package).

### Spatial autocorrelation and historical gene flow

We estimated spatial genetic structure within the five populations previously identified with TESS. Analyses were performed with SPAGeDI 1.3 (Hardy & Vekemans 2002) for each population, using the estimator of kinship coefficient of Nason (Loiselle et al. 1995). Kinship coefficient values (*F*_ij_) were regressed against the natural logarithm, ln(*d*_ij_), to obtain the regression slope, *b*. The spatial positions of individuals were permuted 9999 times to test for the existence of a spatial genetic structure. We compared the extent of spatial genetic structure between sites over the same spatial scale, by calculating *b*_Ld_, the regression slope of *F*_ij_ against ln(*d*_ij_) for *d*_ij_ = 10 km (the maximum distance providing the best representation of pairs of individuals for each population). We then calculated the Sp statistic, defined as Sp* = *−b_(Ld10)_/(1−*F*_N_), where *F*_N_ is the mean *F*_ij_ between neighboring individuals, which was approximated by *F*_(d)_ for the first distance interval (Vekemans and Hardy 2004; Hardy et al. 2006).

### Factors affecting crop-to-wild gene flow and the diversity of flower visitors (fine-scale analyses, France)

All statistical analyses were performed with R version 2.15.3 (R Foundation for Statistical Computing, Vienna, Austria).

#### Anthropogenic factors affecting crop-to-wild introgression

We expected the density of cultivated apple trees to be the principal determinant of recent crop-to-wild introgression rate detected by STRUCTURE (i.e.*, P*_DOM→SYL_). We first investigated the effect of the density of cultivated apple trees (based on apple production in tons/ha/year for the French administrative region concerned) on *P*_DOM→SYL_ in wild apple. We then included cultivated apple density as a fixed factor in the models 1 and 2 described below, as the source of the crop-to-wild gene flow. Because the number and area of apple orchards were highly correlated (*r *=* *0.91, *P *<* *0.001), we investigated the effects of the number and area of apple orchards on *P*_DOM→SYL_ independently in two distinct models (hereafter called models 1 and 2, respectively), controlling for cultivated apple tree density. With this approach, we aimed to determine the relative contributions of orchard number and orchard area to crop-to-wild gene flow in apples. For these analyses, we used the dense sample obtained in France (*N *=* *1028 individuals from 20 sites, Fig. S1), and we ran a generalized linear model (GLM) with a quasi-Poisson distribution, as the data appeared to be overdispersed. Apple production (tonnes/year) and the number of and area covered by apple orchards were extracted, for each French administrative region, from the AGRESTE (http://agreste.agriculture.gouv.fr/).

#### Influence of cultivation and management on the distribution of taxa visiting apple flowers

Anthropogenic effects on introgression rates may also be indirectly mediated by effects on pollinators. An intensification of cultivation and management practices may affect the diversity of flower visitors and, thus, gene flow. We therefore explored the impact of the intensity of cultivation and management on the diversity of the taxa visiting flowers in France (hereafter called model 3). We investigated whether the frequency of apple taxa visiting flowers depended on the intensity of cultivation and management. We ran a GLM with a quasi-Poisson distribution, as the data appeared to be overdispersed. In total, 306 observations corresponding to different intensities of cultivation were recorded (Fig. S1 and Table S3).

The intensity of cultivation and management was obtained from the GLC2000 dataset (The Land Cover Map for Europe in the Year 2000, http://edit.csic.es/Soil-Vegetation-LandCover.html). We defined two classes of cultivation and management intensity, distinguishing between areas with higher and lower intensities of cultivation and management, corresponding to the 1–15 and 17–18 classes of the GLC2000 dataset, respectively. These two classes were defined qualitatively on the basis of the standard classification in the worldwide GLC database as ‘natural’ or ‘cultivated and managed’. Individual geographical coordinates were assigned to these two classes with the extract function (*raster* R package).

The diversity of the insect taxa visiting apple flowers (i.e., the number of observed taxa per insect order) was obtained from a nationwide monitoring exercise based on observations by citizens. Using the database generated by this exercise, entomology experts used the photographs provided to check the preliminary classifications obtained with a simple online key by the citizens participating in the project (for further information on the SPIPOLL project methodology, see Deguines et al. 2012). As it is difficult to differentiate between *M. sylvestris* and *M. domestica* morphologically, we considered that it would not be possible to assign the observations of citizens to one species or the other; these observations were therefore pooled.

## Results

### Crop-to-wild gene flow, population structure, and historical gene flow (large-scale analyses, Europe)

#### Large spatial scale of crop-to-wild gene flow in apple

Failing to take the population structure of the wild species into account can lead to spurious signals of introgression from crop species (Kalinowski 2011). We therefore analyzed the structure of *M. sylvestris* and identified five distinct clusters within the wild species, and in addition a cluster corresponding to *M. domestica* (Fig. S2). The use of *K* values *>6* uncovered no further structure within *M. sylvestris*, indicating that the *M. sylvestris* × *M. domestica* hybrids detected at *K *=* *6 were not artifacts due to genetic structure within the wild apple gene pool. We therefore used cumulative membership coefficient in the five *M. sylvestris* clusters in subsequent analyses to identify crop-to-wild hybrid genotypes.

**Figure 1:**
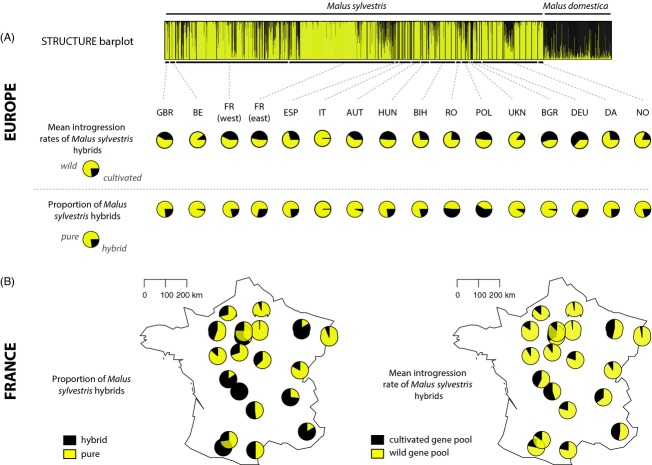
Geographical quantification of introgression rates and numbers of hybrids among European wild apple sites in Europe and, more specifically, in France. (A) STRUCTURE barplot for *K *=* *6, for the European wild apple gene pool (yellow) and the cultivated apple gene pool (black) at the European scale (*N *=* *2228, 66 sites); below, associated country-averaged introgression rates (*P*_DOM→SYL_) and number of hybrids. GB: Great Britain, BE: Belgium, FR (west): Western France, FR (east): Eastern France, ESP: Spain, IT: Italy, AUT: Austria, HUN: Hungary, BIH: Bosnia Herzegovina, RO: Romania, POL: Poland, BGR: Bulgaria, UKN: Ukraine, DEU: Germany, DA: Denmark, NO: Norway. (B) Site-averaged introgression rates (*P*_DOM→SYL_) and number of hybrids in France (*N *=* *1092, 20 sites).

For the total dataset for individuals identified *a priori* as *M. sylvestris* (*N *=* *1889), 436 genotypes (23.1% of the total dataset) showed signs of introgression from *M. domestica* (i.e.*,* cumulative membership coefficients <0.9 and >0.1 in the *M. sylvestris* gene pool, *P*_DOM→SYL_) and 77 genotypes were found to correspond to misidentified *M. domestica* trees (i.e., cumulative membership coefficients <0.1 in the *M. sylvestris* gene pool; 4.1% of the total dataset). The mean introgression rates and numbers of hybrids are presented in Fig.1, Tables S1 and S2. Our results indicated large-scale crop-to-wild gene flow in apple in Europe.

We also observed recent wild-to-crop introgressions into the *M. domestica* cultivars (i.e.*,* cultivars with membership coefficients <0.9 and >0.1 in the *M. domestica* gene pool), as previously demonstrated (Cornille et al. 2012). The mean wild-to-crop introgression rate was 0.15 (Table S3).

#### Spatial genetic variation and population structure in *Malus sylvestris*

For analyzing the population structure of *M. sylvestris*, introgressed individuals (i.e.*,* genotypes assigned to a wild gene pool with a membership coefficient <0.9) were removed. The final dataset included 1376 individuals (62 sites). For each site, the summary statistics for genetic diversity are shown in Table S4. On average, gene diversity was high across sites and across markers [*H*_E_* = *0.79 ± 0.07 (min–max: 0.57–0.89)] and *F*_IS_ values were low [*F*_IS_* = *0.10 ± 0.06 (0–0.21)], although there was a highly significant species-wide heterozygote deficit compared to Hardy–Weinberg expectations (*P *<* *0.001). Mean *F*_ST_ across loci were small [*F*_ST_* = *0.025 ± 0.04 (0–0.56)], but significant for 1349/1831 pairs of sites (*P *<* *0.05). Overall, these results indicate that spatial genetic structure is weak in *M. sylvestris*.

**Figure 2:**
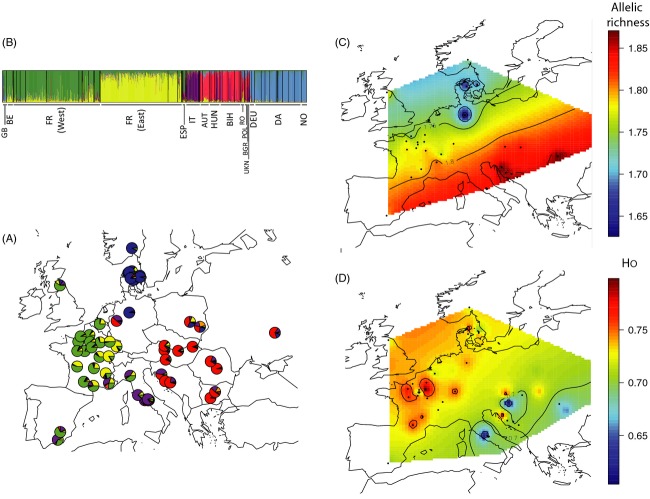
Geographical genetic diversity, genetic structure, and admixture of *Malus sylvestris* in Europe (A) Population structure for the nonintrogressed *Malus sylvestris* dataset (*N *=* *1376, 62 sites), inferred with TESS, for *K*_max_ = 8, showing five distinct genetic clusters. Each vertical line represents an individual. Individuals were grouped by country. Colors represent the inferred ancestry from *K* ancestral populations. For clarity, the 62 sites are grouped by country: GB: Great Britain, BE: Belgium, FR (West): Western France, FR (East): Eastern France, ESP: Spain, IT: Italy, AUT: Austria, HUN: Hungary, BIH: Bosnia Herzegovina, RO: Romania, POL: Poland, BGR: Bulgaria, UKN: Ukraine, DEU: Germany, DA: Denmark, NO: Norway; (B) Map representing the mean membership proportions inferred by TESS for the five detected clusters, for samples of *Malus sylvestris* collected from the same site (62 sites across Europe). (C) and (D) Maps of interpolated allelic richness *A*_R_ and observed heterozygosity *H*_O_, respectively, across Europe.

The map of interpolated *A*_R_ (Fig.2C) showed that genetic variability decreased with increasing latitude, with the highest values found in Southern and Eastern Europe. This latitudinal trend was confirmed by the highly significant negative correlation observed between latitude and *A*_R_ (*r *=* *−0.75, *P ***<*** *0.001). A significant positive correlation between longitude and *A*_R_ was also found (*r *=* *0.33, *P *=* *0.02). The map of interpolated *H*_O_ (Fig.2D) showed a significant negative correlation between longitude and *H*_O_ (*r *=* *−0.30, *P ***=*** *0.03); no correlation between latitude and *H*_O_ was also found (*r *=* *0.19, *P *=* *0.16). This latter map highlights the main admixture zones among *M. sylvestris* populations as regions of high diversity.

Spatially explicit clustering analyses carried out with TESS confirmed the existence of five well-defined clusters within *M. sylvestris* (Figs 2A,B and S3): two of these clusters spanned Western (green) and Central Europe (yellow); one spread over Eastern Europe and the Balkan Peninsula (red); one covered Northern Europe (blue); the last one was located in Italy (purple). DIC values decreased monotonically from *K*_max_ = 2 to *K*_max_ = 9, indicating that increases in the number of clusters continually improved the fit of the model to the data, with a sharper decrease in DIC was observed at *K*_max_ = 8 (Fig. S4) indicating that *K*_max_ = 8 was the best-fit model. However, at *K*_max_ = 8, only five clusters still appeared (Fig. S3), as in three of the eight clusters, the membership coefficients of all individuals were close to 0. We therefore concluded that the biologically most relevant number of clusters was five, as observed in STRUCTURE analyses; we nevertheless used the TESS membership coefficient inferred at *K*_max_ = 8 to define the five populations used in subsequent analyses, as it had the highest DIC value and therefore the best fit to the data. Individuals were assigned to populations for which their membership coefficients exceeded 0.55. The five populations are hereafter referred to as the Western (green, *N *=* *421), Central (yellow, *N *=* *362), Southern (purple, *N *=* *83), Eastern (red, *N *=* *177), and Northern (blue, *N *=* *245) populations. In total, 88 genotypes could not be assigned to any population; *N *=* *1288 individuals were thus retained for population-specific analyses.

For each population, summary statistics for genetic diversity are shown in Table1. Genetic variability (*H*_E_ and *H*_O,_ *A*_R_ and *A*_P_) differed significantly between all populations (Wilcoxon signed rank (WSR) test, *P *<* *0.001) other than the Western and Central populations (WSR, *P *>* *0.60), and the Eastern and Southern populations (WSR, *P *>* *0.44). Overall, the Eastern and Southern populations displayed significantly higher levels of genetic variability, in terms of allelic richness (*A*_R_ and *A*_P_), than the Northern, Western, and Central populations. Higher heterozygosities (*H*_E_ and *H*_O_) were considered to be footprints of recent zones of admixture between different genetic clusters (i.e.*,* the Western/Central populations and Central/Eastern populations).

**Table 1:** Genetic polymorphism, population structure, and the spatial pattern of genetic differentiation in *Malus sylvestris* (*N *=* *1288 individuals)

	Northern	Eastern	Southern	Western	Central
*N*	245	177	83	421	362
*H*_O_	0.66	0.79	0.84	0.72	0.74
*H*_E_	0.72	0.89	0.89	0.78	0.79
*F*_IS_	0.08***	0.11***	0.06***	0.07***	0.07***
*A*_R_	1.72	1.89	1.89	1.78	1.79
*A*_P_	0.67	1.17	1.19	0.73	0.79
**Population structure (colors as in Fig.**2**)**					
*Q*_1_ (blue)	0.92	0.02	0.02	0.01	0.04
*Q*_2_ (red)	0.04	0.85	0.13	0.04	0.02
*Q*_3_ (purple)	0.02	0.11	0.84	0.04	0.05
*Q*_4_ (green)	0.00	0.01	0.01	0.84	0.04
*Q*_5_ (yellow)	0.01	0.01	0.01	0.07	0.86
**Spatial pattern**					
Mean(Ln(dist))	0.53	0.34	1.17	0.77	1.55
*b*	−0.014***	−0.021***	−0.007***	−0.011**	−0.007**
*F*_(1)_	0.04	0.06	0.06	0.06	0.008
*r*^*2*^	0.08	0.15	0.03	0.05	0.005
Sp	0.014	0.022	0.007	0.011	0.007

*N*, sample size of each population; *H*_O_ and *H*_E_, observed and expected heterozygosity, respectively; *F*_IS_, inbreeding coefficient; *A*_R_, mean allelic richness, corrected by the rarefaction method, estimated for a sample size of 2; *A*_P_, private allelic richness, corrected by the rarefaction method, estimated for a sample size of 100; *Q*_1-5_, mean membership coefficient for each of the five clusters inferred by TESS analyses, including hybrids with membership coefficients of up to 0.55 for the cluster concerned; Sp, Sp parameter; mean(Ln(dist)), mean of the logarithm of the geographic distance between genotypes; *b,* slope of the regression between *F*_ij_ and the logarithm of geographic distance; *F*_(1)_, mean *F*_ij_ between individuals from the first distance class; *r*², squared correlation coefficient for the relationship between the logarithm of geographic distance and *F*_i_*;*

** 0.01 < *P *≤* *0.001

*** *P *<* *0.001.

Proportions of admixture differed between the five populations (Fig.2, Tables1 and 2). Substantial admixture was detected between the Western, Southern, and Northern populations, with hybrid numbers highest for the Western and Central populations (*N *=* *321 admixed individuals) and then for the Northern and Western populations (*N *=* *41) (Table2). The Eastern cluster mostly displayed admixture with the Southern (*N *=* *31), Northern (*N *=* *21), and Central (*N *=* *21) populations. The Southern population displayed admixture mostly with the Eastern (*N *=* *31) population and the Western population (*N *=* *23). The admixture detected is indicative of gene flow between these populations. Genetic differentiation (*F*_ST_) was greatest between the Central/Western/Northern and Southern/Eastern populations (Table2). These results indicate a strong west–east differentiation of allelic frequencies, that was detected at *K *=* *2 by TESS and STRUCTURE (Figs S2 and S3).

**Table 2:** Genetic differentiation (upper triangle, *F*_ST_) and total number of admixed genotypes (lower triangle, i.e. individuals assigned to the population with membership coefficients of 0.55–0.9) among the five populations of *Malus sylvestris*

	Northern	Western	Central	Southern	Eastern
Northern	–	0.03	0.06	0.13	0.11
Western	41	–	0.04	0.09	0.08
Central	14	321	–	0.09	0.08
Southern	2	23	11	–	0.04
Eastern	21	13	21	31	–

All *F*_ST_ values were highly significant (*P *<* *0.001).

#### Spatial autocorrelation, isolation by distance (IBD), and historical gene flow

Within each of the five populations, genetic differentiation and geographical distance were significantly correlated, consistent with an IBD model (Table1). The Sp statistic was low suggesting weak spatial genetic structure and high levels of historical gene flow and/or effective population size. Overall, the mean Sp value was 0.01.

### Factors affecting crop-to-wild gene flow and flower visitors (finer scale analyses in France)

#### Anthropogenic factors affecting crop-to-wild introgression

For model 1, the proportion of admixture of the *M. domestica* gene pool into the *M. sylvestris* gene pool (*P*_DOM→SYL_) was found to be dependent on the interaction between cultivated apple density and the number of apple orchards (Table3). Indeed, for small numbers of apple orchards, *P*_DOM→SYL_ were unaffected by cultivated apple density, whereas cultivated apple density had a positive effect on *P*_DOM→SYL_ for large numbers of orchard apple farms. For model 2, *P*_DOM→SYL_ appeared to depend on the interaction between cultivated apple density and the area of apple orchards (Table3) with the same trend found in model 1.

**Table 3:** Summary of minimal models 1, 2, and 3, which were tested for the identification of factors affecting crop-to-wild gene flow and the diversity of visitors to apple flowers

Model	Response variable	Explanatory variable	*F*-value (df)	*P*-value
Model 1	*P*_DOM→SYL_	Cultivated apple density	0.67 (1,563)	0.387
		Number of apple orchards	46.64 (1,563)	<0.001
		Number of apple orchards ^*^ Cultivated apple density	31.53 (1,563)	<0.001
Model 2	*P*_DOM→SYL_	Cultivated apple density	10.01 (1,563)	<0.001
		Area of apple orchards	27.20 (1,563)	<0.001
		Area of apple orchards ^*^ Cultivated apple density	25.92 (1,563)	<0.001
Model 3	Diversity of taxa	Intensity of cultivation and management	125.12 (1,306)	<0.001
		Order	41.97 (1,306)	<0.001

*P*_DOM→SYL_, proportion of admixture of the *Malus domestica* gene pool into the *Malus sylvestris* gene pool; df, degrees of freedom; x^*^y, interaction between explanatory variables *x* and *y*.

#### Factors affecting the diversity of flower visitors

**Figure 3:**
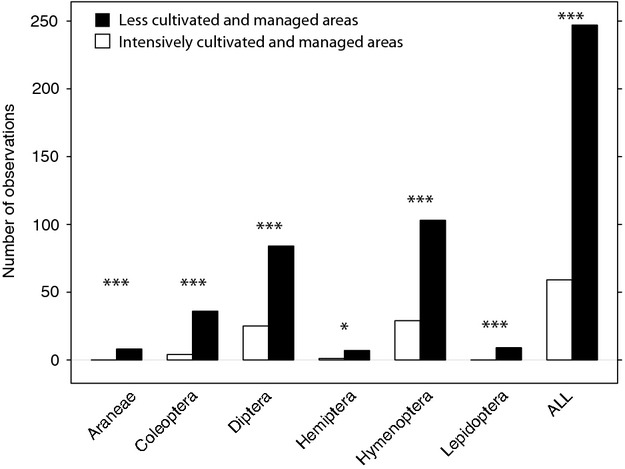
Number of observations for the six orders of insect visitors to apple flowers in France (N = 306), in areas with more intensive (white) and less intensive (black) cultivation and management. ALL: number of observations for all orders considered together. ***: P < 0.0001; *: P < 0.01.

Apple species were visited by 86 taxa from six orders of insects (Fig.3 and Table S5). In model 3, the frequency of taxa visiting apple flowers was significantly lower in intensively cultivated and managed areas (26 taxa, 59 observations) than in less intensively cultivated and managed areas (83 taxa, 247 observations) (Fig.3 and Table3), after controlling for the order to which each taxon belonged. This relationship was also observed for all the insect orders considered (Fig.3 and Table3, *P* < 0.0001).

## Discussion

Previous studies investigating crop-to-wild gene flow in fruit trees have provided estimates of introgression rates, but did not identify the factors influencing these rates (Delplancke et al. 2011; Hoban et al. 2012; Aerts et al. 2013). This study provides new insight into the impact of human activities on the extent of recent crop-to-wild gene flow in fruit trees. We detected (i) large-scale recent introgressions from the cultivated apple to *M. sylvestris*, the extent of which was influenced by the intensity of apple production, and (ii) a decrease in the diversity of apple flower visitors with increasing intensity of cultivation and management practices, suggesting an indirect impact of land-use on pollen flow.

Our study also unraveled the genetic structure of a scattered temperate European fruit tree, through comprehensive sampling. It provides new insight into the history of the wild apple, by revealing the influence of past climatic changes and barriers to dispersal in shaping the genetic structure of this forest fruit tree, resulting in the formation of differentiated genetic clusters in Europe. We also estimated levels of inter- and intracluster gene flow, and we discuss the fundamental and applied impact and significance of these findings below.

### Human impact on crop-to-wild gene flow in apples

Since its introduction into Europe by humans 1500 years ago, the cultivated apple has acted as a source of genes for introgression into the European wild apple (Cornille et al. 2013b). We found that the rates of crop-to-wild introgression varied across Europe and could be accounted for by direct and indirect anthropogenic factors. Interestingly**,** the density of cultivated apple trees, as estimated from apple production, was found to be positively related to recent introgression rates in wild apple populations, with the number of apple orchards surrounding wild populations and with the total orchard area. These results suggest that crop-to-wild introgression in apple is both dependent on the distribution of orchards within the landscape and on their area. It seems intuitive that several large apple orchards scattered across the landscape would act as a more efficient source of crop-to-wild gene flow than a smaller number of small orchards. Our results indicate that human management practices have also had an impact on the insect community visiting apple flowers in France. The numbers of insect orders visiting apple trees were smaller in managed and cultivated areas, suggesting that human disturbance has an indirect impact on gene flow, through negative effects on potential pollinator populations. Further investigations are required to determine the extent to which the changes in apple visitor communities according to land-use affect gene flow from crop-to-wild species and between populations in wild species.

### New insights into the demographic history of the European wild apple

Past climate changes and spatial barriers to dispersal across Europe can explain the population structure of wild apples revealed here. Over the last 2.6 million years, trees species have experienced dynamic alternations between the contraction and expansion of their ranges in Europe, in response to climate changes (Hewitt 1996, 1999; Petit et al. 2003). Our investigations of the population structure and diversity of *M. sylvestris*, based on the use of unprecedented dense sampling in France, provide further insight into the distribution ranges of apples in Europe in the past, over and above that provided by previous studies (Coart et al. 2006; Larsen et al. 2006; Cornille et al. 2013a; Schnitzler et al. 2014). We identified five well-differentiated gene pools with specific spatial distributions, whereas our previous sampling campaign led to the identification of only three clusters (Cornille et al. 2013a). Furthermore, the denser sampling used in this study made it possible to identify hotspots of allelic richness located in the South of France, Italy, the Balkans, and the Carpathian Mountains. The South of France, the Balkans, and the Carpathian Mountains have already been identified as glacial refugia (Cornille et al. 2013a), but our results suggest that Italy and the south-eastern Alps may also have served as glacial refugia for wild apple trees, as already demonstrated for other tree species (Tzedakis et al. 2013). We also found a longitudinal trend for allelic richness that could be potentially accounted for by an east*–*west gradient of climate severity in the Mediterranean region during the last glacial maximum (Conord et al. 2012). Spain would thus have been a minor glacial refugium for *M. sylvestris*, due to its harsher climate. Three main contact zones between clusters were identified, in the South-eastern Alps (between the green and purple clusters), in France (between the green and yellow clusters), and in the Carpathian Mountains (between the red and purple clusters). These results suggest that previous contractions of the distributional range of wild apple during the last glacial maximum in southern Europe were followed by a recolonization of Northern Europe via two main routes: a western front (South of France—Italy, up to Scandinavia) and an eastern front (Balkans—Carpathian Mountains, up to Scandinavia). In particular, the additional Italian cluster detected appears to have acted as a bridge for the recolonization of the western and eastern parts of Europe after glaciation. Barriers to dispersal during recolonization and bottlenecks may also have affected the existing population structure. For instance, populations from northern Germany, Denmark, and Norway have a low allelic richness, and a bottleneck might account for these populations belonging to a distinct gene pool.

### Weak isolation by distance reveals high historical levels of gene flow during the recolonization of Europe

Our findings suggest that, despite the observed population structure, the level of natural intraspecies gene flow during the course of history was high for a wild fruit tree. Indeed, we detected a weak spatial genetic structure within each cluster, spanning large geographic distributions (with low Sp values), suggesting relatively high dispersal capacities and/or effective population sizes (Vekemans and Hardy 2004). Long-distance dispersal events have been reported for another temperate fruit tree, *Sorbus domestica* (Kamm et al. 2009). Nevertheless, we might have expected to find a stronger genetic structure in this zoochorous fruit tree, because fruits carry half-sib or full-sib seeds as dispersal units, rendering dispersal nonrandom. This would be expected to lead to a stronger genetic structure than is observed in wind-pollinated trees (Hardy et al. 2006). However, the spatial genetic structure detected in this study was as weak as that detected in wind-dispersed trees. This may reflect the small number of generations since European recolonization after the last glacial maximum (<10 000 years ago, corresponding to about 1000 generations). Too little time has thus elapsed for genetic differentiation to occur, particularly given the long generation time, overlapping generations and high effective population sizes of trees (Austerlitz et al. 2000). We used genetic distances between individuals to estimate gene flow. This method principally reflects historical patterns of gene flow (Manel et al. 2003). Our estimates may thus not be indicative of local-scale pollen and seed gene flows in contemporary landscapes. Only genetic analyses of parentage (Smouse and Sork 2004) would provide an estimate of the current dispersal abilities of the wild European apple.

## Conclusion and future prospects: a basis for future conservation and breeding programs

The consequences of crop-to-wild gene flow for the wild apple remain unclear, particularly as concerns the possibility of genetic swamping (Lenormand 2002). This phenomenon occurred in the wild ancestor of coconut (*Cocos nucifera*), in which introgression from domesticated populations led to the disappearance of all genetically pure wild individuals (Ellstrand 2014). Crop-to-wild gene flow in apple has been shown to be responsible for a decrease in fitness in populations of the European wild apple in Germany (Bleeker et al. 2007). The European wild apple may also be vulnerable to changes in the diversity of insect visitors to its flowers, resulting in less efficient pollination services, for instance. Given the emblematic nature of the wild apple in Europe and its usefulness as a resource for future breeding programs (Cornille et al. 2014), conservation measures should be urgently considered.

The implementation of conservation programs is challenging, in terms of the application of the recommended practices in the field. Landscape genetics can provide useful information for conservation management, in the form of fundamental knowledge for the identification of high-priority populations for the conservation of genetic variation. Our landscape genetic studies of within-species gene flow and crop-to-wild gene flow in apples in Europe should facilitate the allocation of priority zones for the conservation of the European wild apple, across its natural distribution. We detected five main genetic groups, within which it would be optimal to conserve populations. Southern Europe, housing the main glacial refugia with the highest levels of genetic diversity, should also be considered a priority area for conservation programs, together with France and the Carpathian Mountains, the main suture zones, with their original allelic combinations. The identification of highly introgressed European wild apple populations and of the anthropogenic drivers of crop-to-wild introgressions will also help to dynamically manage these priority conservation zones. This *in situ* conservation could also be combined with the *ex situ* conservation of genetic resources through the creation of gene banks for a core collection of nonintrogressed *M. sylvestris* populations from diverse European regions.

Our landscape genetic investigations would also help to identify the optimal provenance for seeds or germplasm for the reintroduction of wild apples into agroforestry programs (Krauss et al. 2013). Wild apple trees are, indeed, currently being planted in France in the context of agroforestry programs, but the provenance and the degree of introgression of the wild apple germplasm used have never been considered.

In conclusion, the relatively recent introduction of the cultivated apple into Europe, only about 1500 years ago, has particularly facilitated the detection of wild-to-crop and crop-to-wild gene flow and the identification of its determinants. The cultivated-wild apple complex in Europe thus appears to be a good model in which to address both fundamental and applied issues relating to the impact of human landscape changes on gene flow.

## Statement of authorship

AC, PG, and TG conceived and designed the experiments; AC, PG, and TG obtained funding; AC and AF performed the experiments; AC and TG supervised the Master's thesis of AF; AC, AF, and UG analyzed the data. KV and JR provided samples. The manuscript was written by AC with critical input from UG and TG.

## Data accessibility

Data for this study (genetic, visitor flower diversity and relative to apple production) are available at the Dryad Digital Repository: http://doi.org/10.5061/dryad.bm46p.

## References

[b1] Aerts R, Berecha G, Gijbels P, Hundera K, Van Glabeke S, Vandepitte K, Muys B (2013). Genetic variation and risks of introgression in the wild *Coffea arabica* gene pool in south-western Ethiopian montane rainforests. Evolutionary Applications.

[b2] Arnaud JF, Viard F, Delescluse M, Cuguen J (2003). Evidence for gene flow via seed dispersal from crop to wild relatives in *Beta vulgaris* (Chenopodiaceae): consequences for the release of genetically modified crop species with weedy lineages. Proceedings of the Royal Society of London. Series B: Biological Sciences.

[b3] Austerlitz F, Mariette S, Machon N, Gouyon PH, Godelle B (2000). Effects of colonization processes on genetic diversity: differences between annual plants and tree species. Genetics.

[b4] Bleeker W, Schmitz U, Ristow M (2007). Interspecific hybridisation between alien and native plant species in Germany and its consequences for native biodiversity. Biological Conservation.

[b5] Chen C, Durand E, Forbes F, François O (2007). Bayesian clustering algorithms ascertaining spatial population structure: a new computer program and a comparison study. Molecular Ecology Notes.

[b6] Coart E, Vekemans X, Smulders MJM, Wagner I, Van Huylenbroeck J, Van Bockstaele E, Roldán-Ruiz I (2003). Genetic variation in the endangered wild apple (*Malus sylvestris* (L.) Mill.) in Belgium as revealed by amplified fragment length polymorphism and microsatellite markers. Molecular Ecology.

[b7] Coart E, Van Glabeke S, De Loose M, Larsen AS, Roldán-Ruiz I (2006). Chloroplast diversity in the genus *Malus*: new insights into the relationship between the European wild apple (*Malus sylvestris* (L.) Mill.) and the domesticated apple (*Malus domestica* Borkh.). Molecular Ecology.

[b8] Conord C, Gurevitch J, Fady B (2012). Large-scale longitudinal gradients of genetic diversity: a meta-analysis across six phyla in the Mediterranean basin. Ecology and Evolution.

[b9] Cornille A, Gladieux P, Smulders MJ, Roldán-Ruiz I, Laurens F, Le Cam B, Nersesyan A (2012). New insight into the history of domesticated apple: secondary contribution of the European wild apple to the genome of cultivated varieties. PLoS Genetics.

[b10] Cornille A, Giraud T, Bellard C, Tellier A, Le Cam B, Smulders MJM, Kleinschmit J (2013a). Post-glacial recolonization history of the European crabapple (*Malus sylvestris* Mill.), a wild contributor to the domesticated apple. Molecular Ecology.

[b11] Cornille A, Gladieux P, Giraud T (2013b). Crop-to-wild gene flow and spatial genetic structure in the closest wild relatives of the cultivated apple. Evolutionary Applications.

[b12] Cornille A, Giraud T, Smulders MJM, Roldán-Ruiz I, Gladieux P (2014). The domestication and evolutionary ecology of apples. Trends in Genetics.

[b13] Deguines N, Julliard R, de Flores M, Fontaine C (2012). The whereabouts of flower visitors: contrasting land-use preferences revealed by a country-wide survey based on citizen science. PLoS One.

[b14] Delplancke M, Alvarez N, Espíndola A, Joly H, Benoit L, Brouck E, Arrigo N (2011). Gene flow among wild and domesticated almond species: insights from chloroplast and nuclear markers. Evolutionary Applications.

[b15] Ellstrand NC (2003). Current knowledge of gene flow in plants: implications for transgene flow. Philosophical Transactions of the Royal Society of London. Series B, Biological sciences.

[b16] Ellstrand NC (2014). Is gene flow the most important evolutionary force in plants?. American Journal of Botany.

[b17] Ellstrand NC, Prentice HC, Hancock JF (1999). Gene flow and introgression from domesticated plants into their wild relatives. Annual Review of Ecology and Systematics.

[b500] Evanno G, Regnaut S, Goudet J (2005). Detecting the number of clusters of individuals using the software STRUCTURE: a simulation study. Molecular Ecology.

[b18] Feder JL, Egan SP, Nosil P (2012). The genomics of speciation-with-gene-flow. Trends in Genetics.

[b19] Garant D, Forde SE, Hendry AP (2007). The multifarious effects of dispersal and gene flow on contemporary adaptation. Functional Ecology.

[b20] Gross B, Henk A, Forsline P, Richards C, Volk G (2012). Identification of interspecific hybrids among domesticated apple and its wild relatives. Tree Genetics and Genomes.

[b501] Hardy OJ, Vekemans X (2002). SPAGeDI: a versatile computer program to analyse spatial genetic structure at the individual or population levels. Molecular Ecology Notes.

[b21] Hardy OJ, Maggia L, Bandou E, Breyne P, Caron H, Chevallier M-H, Doligez A (2006). Fine-scale genetic structure and gene dispersal inferences in 10 Neotropical tree species. Molecular Ecology.

[b22] Hewitt GM (1996). Some genetic consequences of ice ages, and their role in divergence and speciation. Biological Journal of the Linnean Society.

[b23] Hewitt GM (1999). Post-glacial re-colonization of European biota. Biological Journal of the Linnean Society.

[b24] Hewitt GM (2004). Genetic consequences of climatic oscillations in the Quaternary. Philosophical Transactions of the Royal Society of London. Series B, Biological sciences.

[b25] Hoban SM, McCleary TS, Schlarbaum SE, Anagnostakis SL, Romero-Severson J (2012). Human-impacted landscapes facilitate hybridization between a native and an introduced tree. Evolutionary Applications.

[b26] Jakobsson M, Rosenberg NA (2007). CLUMPP: a cluster matching and permutation program for dealing with label switching and multimodality in analysis of population structure. Bioinformatics.

[b27] Kalinowski ST (2011). The computer program STRUCTURE does not reliably identify the main genetic clusters within species: simulations and implications for human population structure. Heredity.

[b28] Kamm U, Rotach P, Gugerli F, Siroky M, Edwards P, Holderegger R (2009). Frequent long-distance gene flow in a rare temperate forest tree (*Sorbus domestica*) at the landscape scale. Heredity.

[b29] Krauss SL, Sinclair EA, Bussell JD, Hobbs RJ (2013). An ecological genetic delineation of local seed-source provenance for ecological restoration. Ecology and Evolution.

[b30] Kremen C, Williams NM, Aizen MA, Gemmill-Herren B, LeBuhn G, Minckley R, Packer L (2007). Pollination and other ecosystem services produced by mobile organisms: a conceptual framework for the effects of land-use change. Ecology Letters.

[b31] Larsen A, Asmussen C, Coart E, Olrik D, Kjær E (2006). Hybridization and genetic variation in Danish populations of European crab apple (*Malus sylvestris*. Tree Genetics and Genomes.

[b32] Lenormand T (2002). Gene flow and the limits to natural selection. Trends in Ecology & Evolution.

[b33] Loiselle BA, Sork VL, Nason J, Graham C (1995). Spatial genetic structure of a tropical understory shrub, *Psychotria officinalis* (Rubiaceae). American Journal of Botany.

[b34] Manel S, Holderegger R (2013). Ten years of landscape genetics. Trends in Ecology & Evolution.

[b35] Manel S, Schwartz MK, Luikart G, Taberlet P (2003). Landscape genetics: combining landscape ecology and population genetics. Trends in Ecology & Evolution.

[b36] Manning AD, Fischer J, Lindenmayer DB (2006). Scattered trees are keystone structures – Implications for conservation. Biological Conservation.

[b505] Patocchi A, Fernández-Fernández F, Evans K, Gobbin D, Rezzonico F, Boudichevskaia A, Dunemann F (2009). Development and test of 21 multiplex PCRs composed of SSRs spanning most of the apple genome. Tree Genetics & Genomes.

[b37] Petit RJ, Aguinagalde I, de Beaulieu J-L, Bittkau C, Brewer S, Cheddadi R, Ennos R (2003). Glacial refugia: hotspots but not melting pots of genetic diversity. Science.

[b38] Pritchard JK, Stephens M, Donnelly P (2000). Inference of population structure using multilocus genotype data. Genetics.

[b39] Raymond M, Rousset F (1995). GENEPOP (version 1.2): population genetics software for exact tests and ecumenicism. Journal of Heredity.

[b40] Rousset F (2008). Genepop'007: a complete re-implementation of the genepop software for Windows and Linux. Molecular Ecology Resources.

[b41] Schnitzler A, Arnold C, Cornille A, Bachmann O, Schnitzler C (2014). Wild European apple (*Malus sylvestris* (L.) Mill.) population dynamics: insight from genetics and ecology in the rhine valley. Priorities for a future conservation programme. PLoS One.

[b42] Smouse PE, Sork VL (2004). Measuring pollen flow in forest trees: an exposition of alternative approaches. Forest Ecology and Management.

[b43] Storfer A, Murphy MA, Spear SF, Holderegger R, Waits LP (2010). Landscape genetics: where are we now?. Molecular Ecology.

[b44] Szpiech ZA, Jakobsson M, Rosenberg NA (2008). ADZE: a rarefaction approach for counting alleles private to combinations of populations. Bioinformatics.

[b45] Tzedakis PC, Emerson BC, Hewitt GM (2013). Cryptic or mystic? Glacial tree refugia in northern Europe. Trends in Ecology & Evolution.

[b46] Vekemans X, Hardy OJ (2004). New insights from fine-scale spatial genetic structure analyses in plant populations. Molecular Ecology.

